# EpCAM-Independent Enrichment of Circulating Tumor Cells in Metastatic Breast Cancer

**DOI:** 10.1371/journal.pone.0144535

**Published:** 2015-12-22

**Authors:** Helen Schneck, Berthold Gierke, Frauke Uppenkamp, Bianca Behrens, Dieter Niederacher, Nikolas H. Stoecklein, Markus F. Templin, Michael Pawlak, Tanja Fehm, Hans Neubauer, Disseminated Cancer Cell Network (DCC Net) Duesseldorf

**Affiliations:** 1 Department of Obstetrics and Gynecology, University Hospital and Medical Faculty of the Heinrich-Heine University Duesseldorf, Duesseldorf, Germany; 2 NMI Natural and Medical Sciences Institute at the University of Tuebingen, Reutlingen, Germany; 3 Department of General, Visceral and Pediatric Surgery, University Hospital and Medical Faculty of the Heinrich-Heine University Duesseldorf, Duesseldorf, Germany; Witten/ Herdecke University, GERMANY

## Abstract

Circulating tumor cells (CTCs) are the potential precursors of metastatic disease. Most assays established for the enumeration of CTCs so far–including the gold standard CellSearch—rely on the expression of the cell surface marker epithelial cell adhesion molecule (EpCAM). But, these approaches may not detect CTCs that express no/low levels of EpCAM, e.g. by undergoing epithelial-to-mesenchymal transition (EMT). Here we present an enrichment strategy combining different antibodies specific for surface proteins and extracellular matrix (ECM) components to capture an EpCAM^low/neg^ cell line and EpCAM^neg^ CTCs from blood samples of breast cancer patients depleted for EpCAM-positive cells. The expression of respective proteins (Trop2, CD49f, c-Met, CK8, CD44, ADAM8, CD146, TEM8, CD47) was verified by immunofluorescence on EpCAM^pos^ (e.g. MCF7, SKBR3) and EpCAM^low/neg^ (MDA-MB-231) breast cancer cell lines. To test antibodies and ECM proteins (e.g. hyaluronic acid (HA), collagen I, laminin) for capturing EpCAM^neg^ cells, the capture molecules were first spotted in a single- and multi-array format onto aldehyde-coated glass slides. Tumor cell adhesion of EpCAM^pos/neg^ cell lines was then determined and visualized by Coomassie/MitoTracker staining. In consequence, marginal binding of EpCAM^low/neg^ MDA-MB-231 cells to EpCAM-antibodies could be observed. However, efficient adhesion/capturing of EpCAM^low/neg^ cells could be achieved via HA and immobilized antibodies against CD49f and Trop2. Optimal capture conditions were then applied to immunomagnetic beads to detect EpCAM^neg^ CTCs from clinical samples. Captured CTCs were verified/quantified by immunofluorescence staining for anti-pan-Cytokeratin (CK)-FITC/anti-CD45 AF647/DAPI. In total, in 20 out of 29 EpCAM-depleted fractions (69%) from 25 metastatic breast cancer patients additional EpCAM^neg^ CTCs could be identified [range of 1–24 CTCs per sample] applying Trop2, CD49f, c-Met, CK8 and/or HA magnetic enrichment. EpCAM^neg^ dual-positive (CK^pos^/CD45^pos^) cells could be traced in 28 out of 29 samples [range 1–480]. By single-cell array-based comparative genomic hybridization we were able to demonstrate the malignant nature of one EpCAM^neg^ subpopulation. In conclusion, we established a novel enhanced CTC enrichment strategy to capture EpCAM^neg^ CTCs from clinical blood samples by targeting various cell surface antigens with antibody mixtures and ECM components.

## Introduction

CTCs are cancer cells that actively invaded (“motile cells”) or have been shed (“mobile cells”) from the primary tumor into the blood circulation [1]. Therefore, they are considered as cells with metastatic progenitor characteristics and might be useful surrogates for cancer progression and heterogeneity. Indeed, CTCs have been shown to represent a powerful tool to optimize personalized management of metastatic breast cancer. They are of strong clinical value [2–4] and can be assessed as “liquid biopsy” [5] in a fairly easy, fast, and low invasive fashion. It has been estimated that 1g of tumor tissue (10^9^ cells) sheds about 3–4x10^6^ tumor cells into the blood stream per day [6]. Most of these cells may be cleared by first-pass effects or die in the hostile environment of the blood [7], which may–among other factors–contribute to the extreme rarity of CTCs within the peripheral blood flow. Consequently, highly sensitive and specific methods for detection, isolation and molecular characterization in the background of supernumerary blood cell components (1 CTC per 10^6^−10^7^ peripheral mononuclear blood cells) are needed [8–11]. Up to now, various marker-dependent and marker-independent technological advancements have been undertaken for an improved CTC capturing including immunomagnetic, microfluidic and size- as well as function-based methods [12–18]. Marker-dependent approaches using antibodies against EpCAM such as the FDA-approved CellSearch device are dominating recent enrichment strategies. However, considering phenotypic heterogeneity and potential invasion-associated phenotypic plasticity of CTCs, such as epithelial-to-mesenchymal transition (EMT) [19–22] which results in down-regulation of epithelial proteins (including EpCAM), conventional EpCAM-based capturing techniques might miss CTC subpopulations with a more mesenchymal phenotype. Although it has been recently reported that EpCAM-negativity might refer to highly aggressive and invasive CTCs [22, 23], the impact of EMT-like cancer cells on metastatic tumor spread still has to be unraveled. Consequently, to achieve a better understanding of CTC biology in order to overcome treatment failure and to improve disease monitoring/prediction, it is of utmost importance to capture all sorts of CTC subgroups. Thus, to overcome EpCAM-dependence, alternative markers for more comprehensive and efficient CTC detection approaches have to be defined.

Within the presented study, we aimed to improve CTC enrichment/blood testing in an EpCAM-independent manner providing the opportunity to target multiple epithelial- and/or cancer-related antigens expressed on CTCs simultaneously. After intensive literature search we selected several cell surface-specific antibodies (anti-Trop2, -CD49f, -CD146, -CK8, -c-Met, -CD44, -CD47, -AQP5, -ADAM8, -TEM8) [24–30] and components of the extracellular matrix (laminin, collagen I, HA) either immobilized on planar surfaces or coupled to immunomagnetic beads. Efficient cell binding ability was first examined on breast cancer cell lines. In a second step, EpCAM-depleted supernatants comprising potential CTCs that had escaped EpCAM-based selection were used to evaluate our markers in metastatic breast cancer samples. In summary, these fractions were successfully enriched/analyzed for potential EpCAM^neg^ CK^pos^/CD45^neg^ events and array-based comparative genomic hybridization of single cells confirmed the malignant origin of one EpCAM^neg^ subpopulation.

## Material and Methods

### Cell lines and culture conditions

MCF7, SKBR3, HCC1500, ZR-75-1 (all EpCAM^pos^) and MDA-MB-231 (EpCAM^low/neg^) breast cancer cell lines were purchased from the American Type Culture Collection (ATCC, Manassas, VA, US). TMX2-28 cells (EpCAM^pos^) were a generous gift from Prof. K.F. Arcaro (University of Massachusetts, MA, US) [31]. All cell lines were cultured in RPMI 1640 containing 10% (v/v) fetal calf serum and 1% (v/v) penicillin/streptomycin (all Gibco/Life Technologies, Darmstadt, Germany). SKBR3 and ZR-75-1 cells were cultured without any supplements, whereas culture media for MCF7 and TMX2-28 were supplemented with 25 mM HEPES. MDA-MB-231 cells were maintained with supplementation of 2 mM L-glutamine and 20 mM HEPES; for HCC1500 cells 2 mM L-glutamine, 1 mM sodium pyruvate (all Gibco/Life Technologies) and 0.45% (v/v) D-(+) glucose solution (Sigma-Aldrich, Munich, Germany) were added. All cells were cultured at 37°C in a humidified atmosphere with 5% CO_2_. The same culture conditions were fulfilled for cells grown on coverslips in order to check for marker expression by immunofluorescence staining. At approx. 80% confluence, cells were washed once with PBS (Life Technologies) and fixed with ice cold methanol. Until further use, coverslips were stored at -20°C.

### Patient material

Patient samples were gathered within the German DETECT III/IV trials (III: NCT01619111, IV: NCT02035813) wherein patients with primarily HER2-negative metastatic breast cancer are screened for the HER2-status of CTCs (for more information: www.detect-studien.de). Written informed consent was obtained from all participating patients and the study was approved by the Ethical Committee of the Eberhard-Karls University Tuebingen (responsible for DETECT III: 525/2011AMG1) and the local Ethical Committee of the Heinrich-Heine University Duesseldorf (DETECT III: MC-531; DETECT IV: MC-LKP-668). For these patients CellSearch (CS) analysis (Janssen Diagnostics, LLC, Raritan, NJ, US) was routinely performed as described before [32], and additionally, supernatants of EpCAM-depleted fractions were collected. EpCAM-depleted fractions from 25 patients were obtained and processed. Out of these 25, 4 patients donated twice, resulting in a total of 29 analyzed samples.

### Search strategy for cell surface markers

Upfront, a literature search was performed in PubMed (http://www.ncbi.nlm.nih.gov/pubmed), from January 2011 to July 2014, for articles evaluating cell surface markers/ECM molecules for CTC enrichment in breast cancer and other tumor entities using the following keywords: ‘circulating tumor cells’, ‘breast cancer’, and ‘EpCAM negative’. Herein, searches were checked for marker evaluation using commercially available antibodies.

### Cytospin preparation

To test for antigen expression, immunofluorescence staining of fixed cells was performed. For this, cell lines were either spun onto glass slides or cultured on coverslips. Cytospins were prepared as follows: cells were harvested into single cell suspension with 1x StemPro Accutase (Life Technologies), transferred into 15 ml tubes and washed once by centrifugation (1000 rpm, RT, 5 minutes) with subsequent pellet resuspension in PBS. The cell count was determined by adding 10 μl cell suspension to a C-Chip Neubauer improved device (PEQLAB Biotechnologie GmbH, Erlangen, Germany). Afterwards, 5x10^4^ cells/500 μl PBS were spun onto SuperFrost slides (R. Langenbrinck, Emmendingen, Germany) using a ROTOFIX 32 A centrifuge (800 rpm, 2 minutes; Hettich GmbH & Co.KG, Tuttlingen, Germany). Supernatant was removed by aspiration and cytospins were left to dry overnight at RT. Until further use, slides were stored at -20°C.

### Antibodies

The following antibodies were used for immunofluorescence staining and immobilization on surface-modified glass slides (NEXTERION, Schott Technical Glass Solutions GmbH, Jena, Germany) and/or on immunomagnetic beads: anti-pan Cytokeratin-FITC (clone C-11) (GeneTex, Irvine, US), anti-EpCAM (clone Ber-EP4) (Dianova GmbH, Hamburg, Germany), anti-EpCAM (clone VU1D9) (Cell Signaling Technology, Cambridge, UK). Anti-Cytokeratin 8 (clone C51), anti-CD45-Alexa Fluor 647 (clone 35-Z6) and anti-ADAM8, rabbit polyclonal (cat. no. sc-25576) were all obtained from Santa Cruz Biotechnology, Dallas, TX, US. Anti-CD49f (clone GoH3), anti-CD146 (clone N1238), anti-TEM8, rabbit polyclonal (cat. no. ab21270) and rat IgG2a, kappa monoclonal (clone RTK2758) were acquired from Abcam, Cambridge, UK. Anti-Trop2 (clone 162–46) and mouse IgG1 isotype control (clone MOPC-21) were purchased from BD Pharmingen, Heidelberg, Germany. Anti-CD47, sheep polyclonal (cat. no. AF4670), anti-HGF R/c-MET (clone 95106) and isotype controls for rabbit (cat. no. AB-105-C) and sheep (cat. no. 5-001-A) IgG were supplied by R&D Systems, Minneapolis, MN, US. Anti-CD44, rabbit polyclonal (cat. no. HPA005785) was from Sigma-Aldrich, Munich, Germany.

For immunofluorescent labeling of cells on cytospins or grown on coverslips, respectively, the following secondary antibodies were used: Alexa Fluor 488 Goat Anti-Mouse IgG (H+L) Antibody, Alexa Fluor 488 Goat Anti-Rabbit IgG (H+L) Antibody, Alexa Fluor 594 Goat Anti-Mouse IgG (H+L) Antibody, Alexa Fluor 594 Goat Anti-rabbit IgG (H+L) Antibody, Alexa Fluor 594 Goat Anti-rat IgG (H+L) and Alexa Fluor 488 Donkey Anti-Sheep IgG (H+L) Antibody (all Life Technologies).

### Immunofluorescence

Cytospins were thawed and fixed with PBS/4% (v/v) paraformaldehyde for 10 minutes at RT. Slides were then washed three times for 3 minutes with 1x Wash Buffer (Dako, Hamburg, Germany). Cells on cytospins as well as cells grown on coverslips were permeabilized with PBS/0.1% (v/v) Triton-X-100 for 10 minutes on ice and washed three times. In case of secondary antibody application, one drop of protein-block (serum-free; Dako) was applied, incubated for 30 minutes in a humid chamber and decanted. Primary antibody incubation (1:100–1:50) was performed for 1 hour at RT. After three washing steps, cells were incubated with secondary fluorescent antibodies (1:200) together with DAPI (1 μg/ml; Sigma-Aldrich) for 30 minutes. All antibodies were diluted in Antibody Diluent (Dako). Slides/coverslips were washed again and finally mounted with Fluorescence Mounting Medium (Dako). Samples were stored at 4°C until imaging with an Axioplan 2 microscope (Zeiss, Goettingen, Germany).

### Generation of single- and multi-marker microarrays

For cell adhesion experiments on planar surfaces, single- and multi-marker microarrays comprising different capturing molecules (antibodies and ECM molecules, see S1 Table and S1 Fig) were produced. Herein, different surface-functionalized NEXTERION coated glass slides (Aminosilane, Epoxysilane, Nitrocellulose, Hydrogel, and Aldehydesilane (AL); Schott Technical Glass Solutions GmbH, Jena, Germany) were available and tested for our application. Initially, 5x5 spot arrays with 16 arrays per slide were generated (spotting molecules and array layout see S1 Fig). All reagents were diluted in PBS to a final concentration of 0.2 mg/ml and spotted onto the slides, whereas 20 single-droplets of 0.4 nl volume per reagent and spot were deposited using a piezoelectric non-contact print robot (NanoPlotter 2, GeSiM, Grosserkmannsdorf, Germany). The spot diameter was about 500 μm with a spot-to-spot distance (pitch) of 1 mm. After spotting, the slides were blocked with StabilGuard Immunoassay Stabilizer (SurModics, Eden Prairie, MN, US), thoroughly washed with double-distilled water, dried and stored at 4°C in the dark until further use. Optimal antibody concentrations for extended multi-marker array generation were determined. Therefore, purified anti-EpCAM [Ber-EP4], anti-Trop2 and anti-CD49f antibodies were titrated in eight dilutions (0.2, 0.1, 0.05, 0.025, 0.01, 0.005, 0.002, 0.0 mg/ml) in duplicates and spotted manually onto NEXTERION slides AL, resulting in 16 spots per slide and antibody. A single spot consisted of a 1 μl-droplet producing a diameter of approx. 2 mm. Slides were blocked and stored as described above. The final multi-marker array layout consisted of 36 (6x6) spots placed in a total area of 5x5 mm^2^. The spot diameter was about 500 μm with a pitch of 800 microns. Therein, anti-CD49f, anti-Trop2, anti-EpCAM, laminin, collagen I and HA spots were immobilized in both, individually and in combination (antibody-antibody, antibody-ECM, ECM-ECM). After printing, the arrays were handled as described above. Immediately, prior to cell incubation, slides were washed three times for 5 minutes with PBS, disposable fluid chambers (custom-designed by STRATEC Biomedical AG, Birkenfeld, Germany) were attached to completely dried slides and arrays were equilibrated with 300 μl RPMI1640/25 mM HEPES for 2 minutes at RT.

### Cell adhesion on single- and multi-marker arrays

Cell adhesion experiments on single- and multi-marker arrays were performed with a pool of EpCAM^pos^ cells (MCF7, SKBR3, HCC1500, TMX2-28 and ZR-75-1) in comparison to MDA-MB-231 (EpCAM^low/neg^). EpCAM^pos^ cell lines were pooled in order to assure functionality of applied capture approaches.

#### Cell adhesion on manually spotted single-marker arrays

Slides with manually spotted antibodies were washed three times with PBS and were then assembled with ProPlate multi-array chambers (16-Square Well; Grace Bio-Labs, Bend, US) according to manufacturer’s instructions. Each cavity was filled with 2.5x10^4^ cells (either cell pool or MDA-MB-231) adjusted to 250 μl with RPMI1640/25 mM HEPES and cell adhesion was analyzed after incubation for 2 hours at 37°C and 600 rpm. Supernatants were removed and cavities were washed once with PBS (+MgCl_2_/CaCl_2_). Bound cells were visualized by staining with 0.05% w/v Coomassie Brilliant Blue (10 minutes; and 3x5 minutes PBS wash) and subsequent imaging with a Nikon Eclipse TE2000-U microscope (Nikon GmbH, Duesseldorf, Germany).

#### Cell adhesion on multi-marker microarrays

To discriminate both cell populations (EpCAM^pos^ *vs* EpCAM^low/neg^) after adhesion, 2x10^5^ cells were stained with either 1 μM MitoTracker Green FM (cell pool) or MitoTracker Orange CM (MDA-MB-231) (Molecular Probes/Life Technologies) for 45 minutes at 37°C (protected from light). Afterwards, cells were washed with RPMI1640/25 mM HEPES and 300 μl cell suspension (EpCAM^pos^ plus EpCAM^low/neg^) was applied for array incubation onto prepared NEXTERION AL slides. Incubation of cells lasted for 2 hours at 37°C with horizontal shaking (450 rpm). After incubation, disposables were rinsed twice, then removed and slides were mounted with 20 μl VECTASHIELD Mounting Medium containing DAPI (Vector Laboratories, Burlingame, CA, US) and were finally covered for imaging with a BIOREVO BZ-9000 fluorescence microscope (KEYENCE, Neu-Isenburg, Germany). Total cell fluorescence was quantified with the ImageJ/Fiji 1.46 software [33].

### Immunomagnetic bead coating and cell capture

Positive selection of breast cancer cell lines as well as of potential tumor cells within the EpCAM-depleted sample fractions was achieved by employing immunomagnetic enrichment with either Dynabeads (Life Technologies) or Bio-Adembeads (Ademtech, Pessac, France) coated with antibodies or HA. Dynal MPC-S/MPC-L (Life Technologies) magnets were used for magnetic separation of beads.

#### Direct coating of Dynabeads and Bio-Adembeads with antibodies

Antibodies were coupled to Dynabeads goat anti-mouse IgG, Dynabeads sheep anti-rat IgG and Dynabeads M-280 sheep anti-rabbit IgG according to the manufacturer’s protocol. Briefly, after pre-washing the beads with 1 ml PBS/2 mM EDTA/1% (v/v) FCS (isolation buffer), 25 μl (1–1.75x10^7^) beads/reaction were incubated with 0.5 μg primary capture antibody for 45 minutes at 4°C while gently tilting and rotating. Afterwards, coated Dynabeads were washed twice in 1 ml isolation buffer, resuspended in the initial buffer volume and stored at 4°C until further use. Additionally, Bio-Adembeads goat anti-mouse IgG and Bio-Adembeads goat anti-rat IgG were used. Coupling conditions were adjusted to those for Dynabeads with incubation, buffer and storage conditions being in accordance with the aforementioned procedure.

#### Direct coating of Dynabeads with hyaluronic acid

Hyaluronic acid (HA, from *Streptococcus pyrogenes*, Calbiochem/Merck Millipore, Darmstadt, Germany) was coupled to Dynabeads MyOne Carboxylic Acid (Life Technologies). Therefore, 3 mg beads were used for coating with 150 μg HA. HA concentration was adjusted to 2.5 mg/ml in 25 mM 2-(*N*-morpholino)ethanesulfonic acid (MES; Sigma-Aldrich) buffer, pH 6. Beads were washed twice with 300 μl 25 mM MES, pH 6 for 10 minutes whilst rotating. For bead activation 50 μl 1-Ethyl-3-[3-dimethylaminopropyl] carbodiimide hydrochloride (EDC; Thermo Fisher Scientific, Rockford, US) and 50 μl *N*-hydroxysulfosuccinimide (sulfo-NHS; Thermo Scientific), diluted in 25 mM MES (pH 6) to a concentration of 50 mg/ml, were added and incubated for 30 minutes at RT and 650 rpm. Supernatant was discarded and beads were washed twice as described before. For ligand immobilization, HA was added to the activated beads in a total volume of 100 μl and after mixing thoroughly, incubated for 30 minutes at RT and 450 rpm. Supernatant was removed and the beads were incubated with 300 μl 50 mM Tris, pH 7.4 (Sigma-Aldrich) for 15 minutes at RT to quench the non-reacted activated carboxylic acid groups. Beads were washed four times with 50 mM Tris, pH 7.4 and were finally resuspended in PBS/0.02% (w/v) NaN_3_ to their original volume. Functionalized beads were stored at 4°C until further use. Prior to sample incubation, beads were washed twice with isolation buffer.

#### Cell capture with Dyna/Adembeads

For each capture reaction, 25 μl (1x10^7^)/15 μl coated Dyna/Adembeads were incubated with 2.5x10^5^ cells for 30 minutes at RT and tilt rotation. Bead fractions were washed three times, resuspended in 500 μl PBS and transferred to 1.5 ml Protein LoBind tubes (Eppendorf AG, Hamburg, Germany). For imaging, bead/cell suspensions were mounted with 20 μl VECTASHIELD Mounting Medium/DAPI onto microscope slides.

### Processing of EpCAM-depleted breast cancer clinical samples and detection of recovered cells

To enrich potential CTCs that had not been captured by the EpCAM-positive selection through the CS system, EpCAM-depleted fractions were further analyzed via immunomagnetic beads coated with specific cancer-related markers. All fractions were preserved by the addition of 300 μl 0.1236 M EDTA for overnight storage at 4°C. For each collected sample fraction (21–39 ml), 2 to 6 separate enrichment approaches were carried out. Whole fraction volumes were equally separated and transferred to 15 ml-tubes. Isolation buffer was added to a final volume of 10 ml. Then samples were centrifuged (600xg, 10 minutes, RT) and the buffer/plasma fraction was removed. Prior to bead incubation, the remaining white/red blood cell fraction was recompleted to 5 ml. For incubation with single bead preparations (antibody or ECM) 25 μl (1x10^7^)/15 μl pre-coated Dyna/Adembeads were added, for combined Dynabead approaches (antibody plus antibody/ECM) 12.5 μl each were mixed. Positive cell isolation was obtained as described above. Previous to staining of captured cells, bead/cell suspensions were pre-incubated with 5 μl Human TruStain FcX (Biolegend, San Diego, US) for 5 minutes. Samples were then stained with anti-pan-CK-FITC (1:400), anti-CD45-Alexa Fluor 647 (1:20) and DAPI (1 μg/ml) adjusted to 100 μl with PBS/0.1% Tween 20 for 30 minutes at 4°C and 450 rpm. Beads were washed twice in PBS, deposited onto microscope slides and mounted in Fluorescence Mounting Medium (Dako). Slides were stored at 4°C until imaging with an Axioplan 2 fluorescence microscope (Zeiss, Goettingen, Germany) considering CK^pos^/CD45^neg^/DAPI^pos^ stain criteria.

### Isolation of single cells and molecular characterization of CTCs

#### Single cell isolation

Single cells were isolated via the CellCelector (CC) device (ALS, Jena, Germany), an automated robotic micromanipulator system, to perform subsequent genomic analysis. Bead-enriched and stained samples from the EpCAM-depleted fractions were screened for CK^pos^/CD45^neg^ events with the CC, whereas EpCAM^pos^ CTCs were selected and identified via CS prior to re-identification with the CC. For improved re-identification of EpCAM^pos^ CTCs with the CC, cartridges were re-inserted into the MAGNEST holder, incubated for 15 minutes, and supernatants (approx. 320 μl) were discarded before the staining solution (DAPI (1 μg/ml), anti-CD45-Alexa Fluor 647 (1:20) in 300 μl PBS) was added. Cells were re-stained for 4 hours at 4°C in the dark. After magnetic incubation (15 minutes), the staining solution was removed and cells were washed with 300 μl PBS. Cartridge contents were mixed thoroughly ensuring that all cells dislodged from the cartridge wall, and were transferred onto glass slides for CC analysis. All samples (CS and bead-enriched) were screened for a positive DAPI/CK and a negative CD45 stain and single cells were deposited within 10 μl-droplets of PBS into 0.15-ml PCR tubes. Tubes were centrifuged for 10 minutes at 1300 rpm and 9 μl of PBS were removed. Finally, cells were frozen at -80°C until further processing.

#### Whole genome amplification

Whole genomes of isolated single cells were amplified using the Ampli1 whole genome amplification (WGA) Kit (Silicon Biosystems, Bologna, Italy) according to the manufacturer’s instructions. Herein, the one-day protocol with an extended overnight step for cell lysis was performed. The quality of the Ampli1 WGA output product was evaluated by running the Ampli1 QC Kit (Silicon Biosystems), which assays a multiplex PCR of four markers. Two μl of the WGA product were analyzed according to the manufacturer’s protocol. Samples displaying three or four bands in the multiplex PCR were used for array-based comparative genomic hybridization (aCGH) analysis [34].

#### Array-based comparative genomic hybridization and data analysis

aCGH (4×180k arrays, Agilent Technologies, Waldbronn, Germany) was performed as previously described [34–36] using 1 μg of the primary amplification product. As a reference we used WGA products of single GM14667 cells, a cell line derived from normal male B-lymphocytes. For data analysis the output image files were imported, normalized and the fluorescent ratios for each probe were determined using the Feature Extraction software (Agilent Technologies, Version 10.7.3.1, Protocol CGH_1105_Oct09). Data visualization and analysis was performed with the Genomic Workbench 6.5.0.18 software using the ADM-2 algorithm with a threshold of 6.0, centralization by legacy with a threshold of 4.0 and a bin size of 10.

## Results

### Evaluation and expression of capture cell surface proteins

To successfully capture EpCAM-negative or -low breast cancer cells, different antibodies specific for cell surface proteins (Trop2, CD49f, CD146, CK8, c-Met, CD44, CD47, AQP5, ADAM8, TEM8) as well as ECM molecules (laminin, collagen I, HA) were tested on breast cancer cell lines with different EpCAM expression levels. These proteins were selected upon literature-based research and were partly described previously with regard to tumor cell enrichment [24–30].

**Fig 1:**
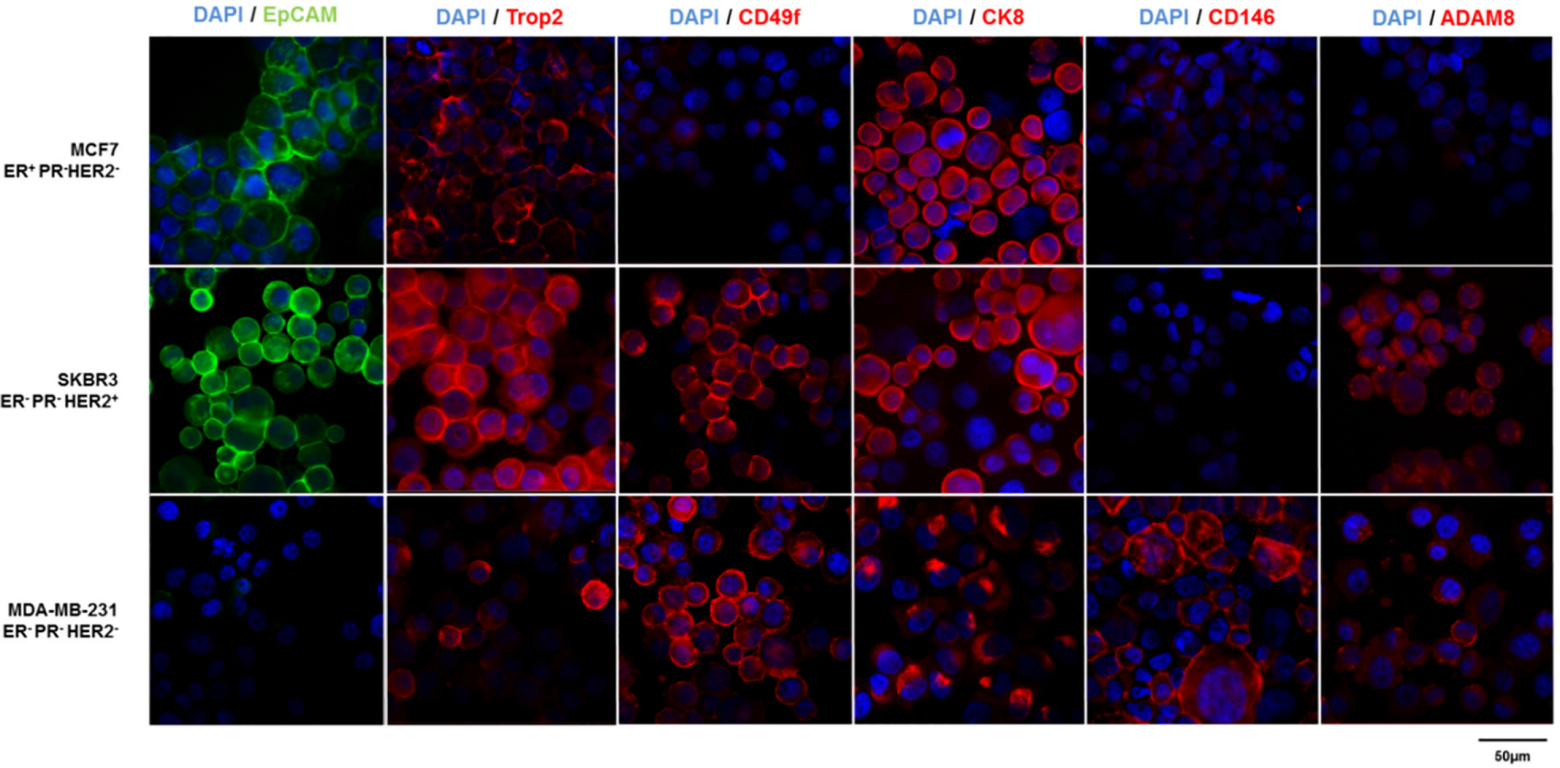
Expression of surface markers on EpCAM^pos^ and EpCAM^low/neg^ cells. Differential protein expression of EpCAM (green), Trop2, CD49f, CK8, CD146 and ADAM8 (red) in EpCAM^pos^ (MCF7, ER^+^PR^-^HER2^-^, top; and SKBR3, ER^-^PR^-^HER2^+^, middle) and EpCAM^low/neg^ (MDA-MB-231, ER^-^PR^-^HER2^-^, bottom) cell lines is displayed by immunofluorescence staining of cytospins; blue = DAPI, 40x magnification.

All studied proteins were expressed heterogeneously and to varying degrees in the examined EpCAM^pos^ (MCF7/SKBR3) and EpCAM^low/neg^ (MDA-MB-231) cell lines (Fig 1), with CD49f and CD146 showing the most abundant expression in the EpCAM^low/neg^/basal-like cell line MDA-MB-231. Regarding the expression of Trop2, CD49f as well as ADAM8 within the EpCAM^pos^ cell lines, HER2-amplified SKBR3 cells revealed more abundant expression levels compared to MCF7 (luminal A subtype). Furthermore, protein distribution throughout the cells for CK8 and CD146 differed greatly. For instance, signals for CD146 exhibited membranous and cytoplasmic localization within MDA-MB-231 cells, whereas it was expressed almost exclusively in the nucleus within luminal B subtype cells (e.g. HCC1500, data not shown).

### Cell enrichment using single-/multi-marker arrays and immunomagnetic beads

**Fig 2:**
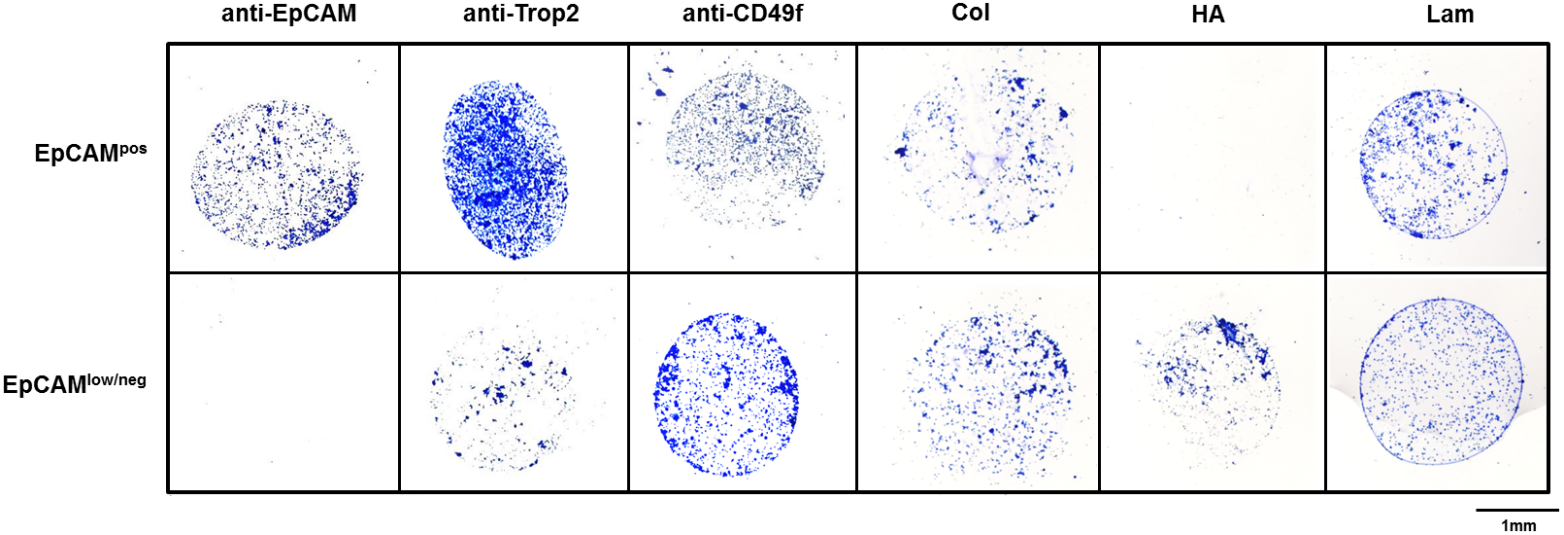
Adhesion of EpCAM^pos/neg^ cells on manually spotted arrays. 2.5x10^4^ EpCAM^pos^ cells (pool of MCF7, SKBR3, HCC1500 and ZR-75-1; upper panel) and EpCAM^low/^_neg_ cells (MDA-MB-231; lower panel) were incubated for cell adhesion experiments on glass substrates (NEXTERION slides AL) manually coated with anti-EpCAM [Ber-EP4], anti-Trop2, anti-CD49f (0.1 mg/ml each), collagen I (Col), hyaluronic acid (HA) and laminin (Lam) (0.2 mg/ml each). Cell adhesion was visualized by Coomassie; 20x magnification.

Based on previous tests of various functional substrates on NEXTERION coated glass slides, the AL coating was chosen since it provided the most specific binding of cells on capture spots and low background (S1 Fig). In subsequent titration experiments, the optimal spotting concentration of antibodies/ECM molecules was reduced to 0.1 mg/ml leading to maximum cell adhesion as visualized by a positive Coomassie stain. Nonspecific binding to the AL substrate itself (pure spotting buffer) as well as to an isotype control (mouse) or bovine serum albumin could not be observed at any time on single marker arrays (S1 Fig). In case of absent cell binding despite a positive immunofluorescence staining on cytospins—observed for AQP5, CK8 and CD146—successful antibody coupling to the substrate was examined after visualization with AlexaFluor 594. Staining of spotted arrays revealed that coupling with anti-CD146 was unsuccessful, whereas anti-CK8 and anti-AQP5 antibodies bound to the slides, but were non-functional. Examples for successful cell adhesion of EpCAM^pos^ (cell pool of MCF7, SKBR3, HCC1500 and ZR-75-1) and EpCAM^low/neg^ (MDA-MB-231) cells to anti-EpCAM, anti-Trop2, anti-CD49f and three ECM molecules (collagen I, HA, laminin) are representatively shown in Fig 2.

Except for HA, all investigated molecules could capture the EpCAM^pos^ cell pool. According to the Coomassie blue signal, cell adhesion to Trop2 and EpCAM spots was most abundant, however binding to CD49f and collagen/laminin could also be observed. In contrast, MDA-MB-231 cells adhered to Trop2, CD49f and all three ECM spots, while the EpCAM spot did not reveal any cell attachment; and capturing through anti-CD49f and HA, MDA-MB-231 binding was much more prominent compared to the EpCAM^pos^ pool.

In continuation of the initial cell adhesion experiments, single arrays were then extended to 36-spot arrays including further antibodies (CD44, CD47, c-Met, TEM8), isotype controls and marker combinations (ab/ab; ab/ECM; ECM/ECM) for anti-CD49f, anti-Trop2, anti-EpCAM, laminin, collagen I, and HA. Spotted capture molecules are listed in S1 Table.

**Fig 3:**
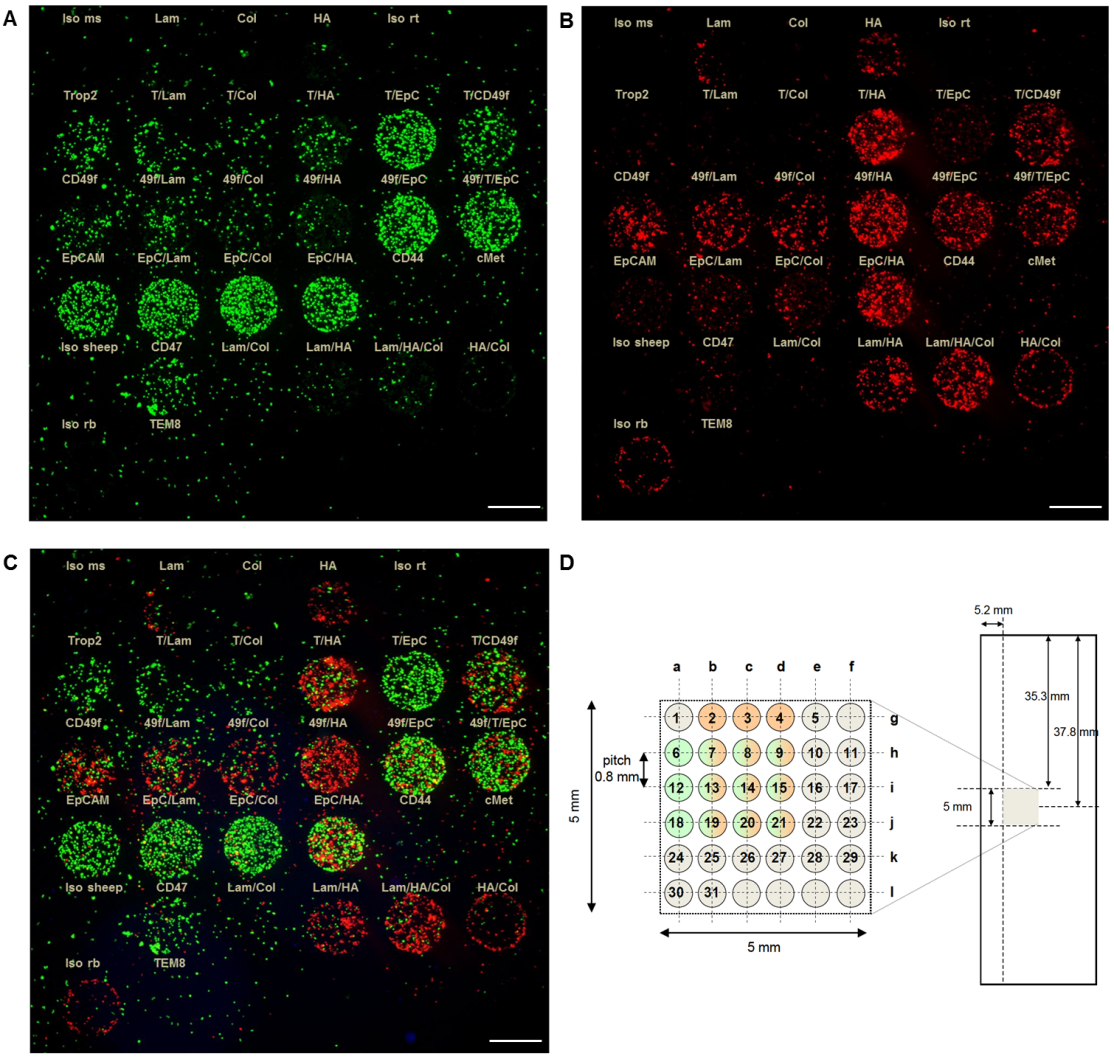
Adhesion of EpCAM^pos/neg^ cells on multi-marker arrays. 2x10^5^ EpCAM^pos^ (cell pool of MCF7, SKBR3, HCC1500, ZR-75-1, TMX2-28) (A) and EpCAM^low/neg^ cells (MDA-MB-231) (B) were either stained with 1 μM MitoTracker Green FM (A) or MitoTracker Orange CM (B) and incubated for cell adhesion experiments on NEXTERION slides AL, coated with different antibodies and ECM molecules, alone and in combination (0.1 mg/ml each). The labeling above the single spots indicates respective capture molecules (Iso = isotype control, ms = mouse, Lam = laminin, Col = collagen, HA = hyaluronic acid, rt = rat, T = Trop2, EpC = EpCAM, 49f = CD49f, rb = rabbit). (C) Overlay image of (A) and (B); scale bars (white) = 500 μm, 20x magnification. (D) Array layout (5x5 mm) with 36 spots (spot diameter = 500 μm; pitch = 800 μm) printed on NEXTERION slides AL.

To distinguish and quantify bound EpCAM^pos/neg^ populations, cells were pre-stained with either MitoTracker green (EpCAM^pos^) or MitoTracker orange (EpCAM^neg^) (Fig 3). Total cell fluorescence (integrated density) for each single spot was quantified using the ImageJ/Fiji 1.46 software. Herein, fluorescence signals per spot are displayed as mean values of three individual experiments (S2 Fig). EpCAM^pos^ cells adhered to all spots containing EpCAM antibody (Fig 3A, 3C and 3D: spots 10, 16–21; S2 Fig). Moreover, binding to anti-EpCAM was increased when it was combined with an ECM molecule or anti-Trop2 or anti-CD49f antibodies. Less binding was observed to spots comprising anti-Trop2 (spots 6–9), CD49f (spots 12–15) and CD47 (spot 25) and almost no adhesion to ECM-alone and isotype control (spots 1, 5, 24, 30) spots was obtained.

In contrast, binding of EpCAM^low/neg^ MDA-MB-231 cells revealed a different pattern (Fig 3B–3D and S2 Fig): strong adhesion to anti-CD49f and HA could be observed—both alone (spots 4, 12) and in combination with laminin and collagen (spots 9, 11, 13–17, 21, 27–29). However, only marginal binding for Trop2 and EpCAM spots (spots 6–8, 10, 18–20) was detectable. A slight signal deriving from cells bound to the rabbit isotype control (spot 30) could be identified. Cell adhesion for both populations to spots coated with CD44 (spot 22), c-Met (spot 23) and TEM8 (spot 31) antibodies was almost undetectable (Fig 3C).

After successful testing, surface enrichment protocols in single- and multi-arrayed formats were changed to immunomagnetic bead-based enrichment in order to establish an approach more suitable for clinical samples—first using Dynabeads and later including Adembeads. Adembeads were more compatible with subsequent microscopic analysis (e.g. lower autofluorescence) and cells could easily be distinguished from the beads.

**Fig 4:**
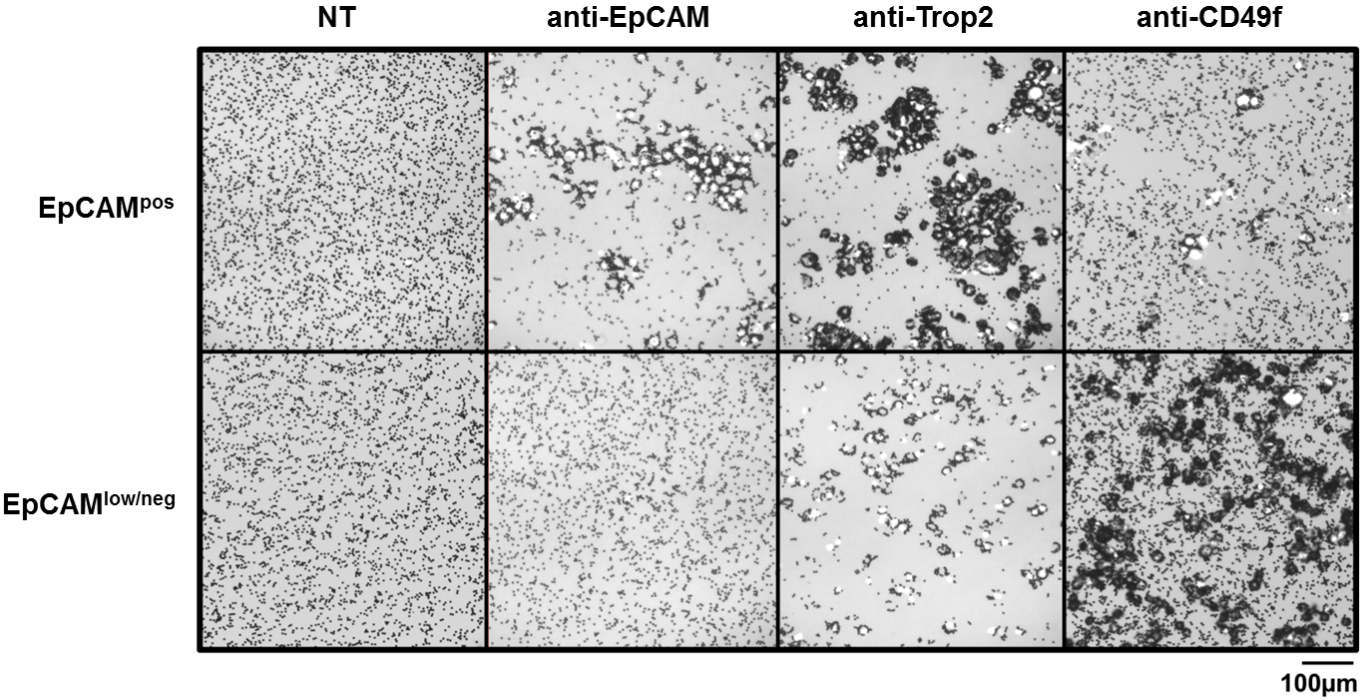
Enrichment of EpCAM^pos/neg^ cells with Dynabeads. EpCAM^pos^ (upper panel; MCF7, SKBR3, T47D, HCC1500 and ZR-75-1) and EpCAM^low/neg^ (lower panel; MDA-MB-231) cells were captured with Dynabeads coupled with antibodies for EpCAM, Trop2 and CD49f (1 μg ab/2.5x10^5^ cells/1x10^7^ beads). No cells bound to uncoated Dynabeads (NT = no target). Cells were imaged after a DAPI stain and are depicted as brightfield/DAPI merge; 10x magnification.

All IgG-coated Dyna-/Adembead preparations, both uncoupled and coupled to antibodies, were tested for their cell binding ability. Representative capturing upon coupling to anti-EpCAM, anti-Trop2 and anti-CD49f of pooled EpCAM^pos^ as well as EpCAM^low/neg^ cells is displayed in Fig 4. EpCAM^pos^ cells (Fig 4, upper panel) can be recovered by anti-EpCAM and anti-Trop2 bead preparations and, to a lesser extent by anti-CD49f. MDA-MB-231 cells (Fig 4, lower panel) can be efficiently enriched with beads coupled to anti-CD49f. Besides that, nonspecific binding of both cell populations to no-target bead controls could not be detected.

### Enrichment of CTCs from EpCAM-depleted breast cancer clinical samples

**Fig 5:**
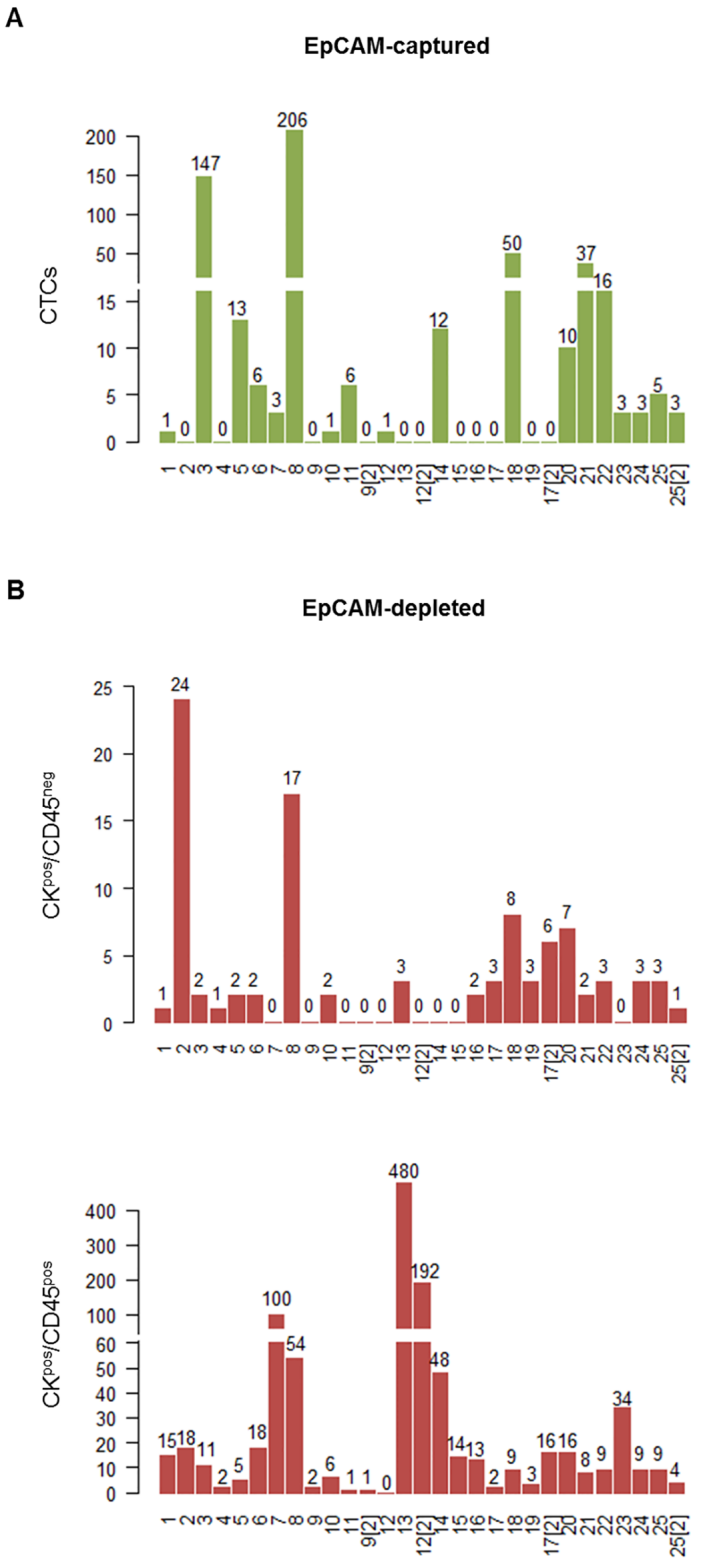
Number of EpCAM-enriched CTCs and potential CTCs/double positive events (CK^pos^/CD45^pos^) within the EpCAM-depleted fractions in blood of breast cancer patients. (A) CTC count determined by EpCAM-enrichment and subsequent CK/DAPI stain in 29 blood samples of 25 patients (DIII and DIV). (B) Number of potential CTCs (top) and double positive events (bottom) within the respective EpCAM-depleted supernatants of the same blood samples; total event numbers (CK^pos^/CD45^neg^ and CK^pos^/CD45^pos^) represent the sum of all events identified after 2–6 immunomagnetic enrichments for each blood/patient sample.

**Fig 6:**
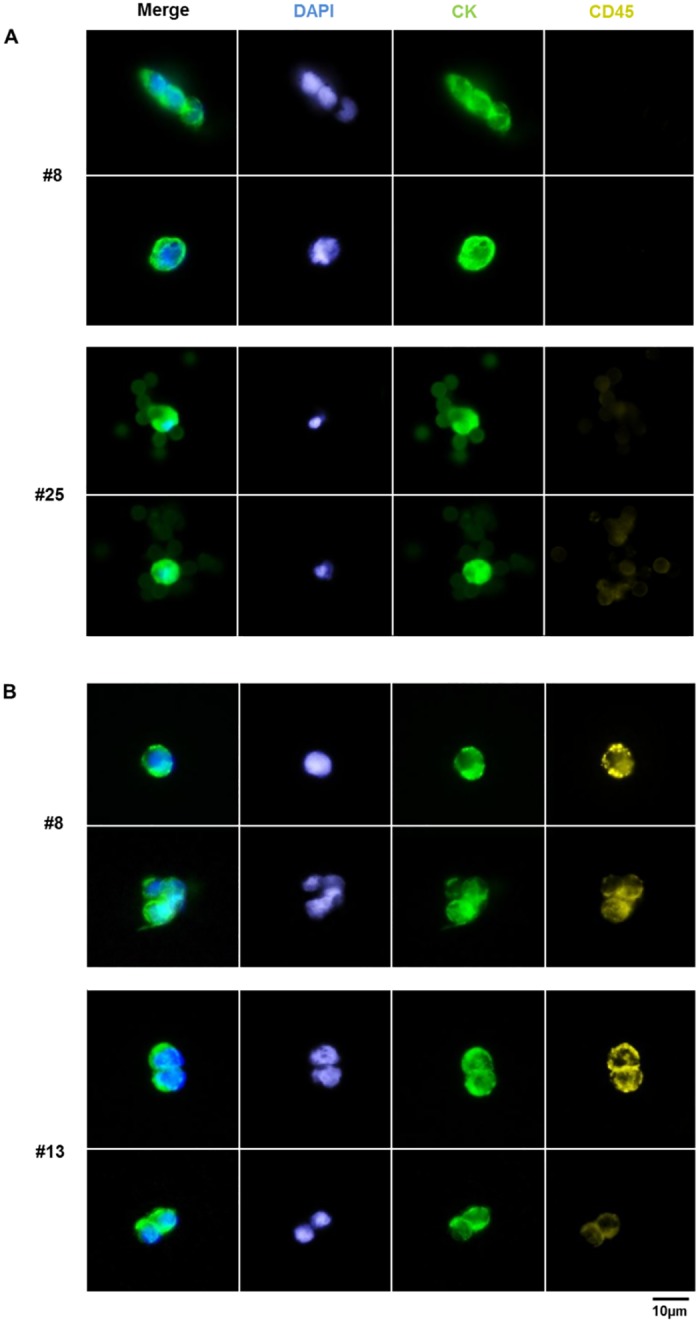
Representative images of potential CTCs (CK^pos^/CD45^neg^) and double positive (CK^pos^/CD45^pos^) events enriched from EpCAM-depleted patient samples. Immunofluorescence staining of (A) CTCs (top: #8, Adem-c-Met; bottom: #25, Dyna-CD49f) and (B) CK^pos/^CD45^pos^ events (top: #8, Adem-c-Met; bottom: #13, Adem-Trop2) enriched after EpCAM-depletion are shown. Adem-/Dynabeads captured cells were stained for DAPI (blue), pan-CK (green) and CD45 (yellow); 40x magnification.

Blood samples from 25 breast cancer patients (Table 1) were analyzed by CellSearch for EpCAM^pos^ CTCs: 11 out of 29 blood samples from 25 patients were negative for CTCs (8x) or CTC count could not be determined (3x); three samples had 1 CTC and 15 samples harbored at least 3 CTCs (mean: 20, median: 3; range 0–206 CTCs) (Fig 5A; Table 1). Additionally, to apply the established workflow, respective EpCAM-depleted fractions for all samples were collected and afterwards processed with beads coupled to antibodies against either Trop2, CD49f, CK8, CD44, ADAM8, CD146, c-Met or to HA, alone or in combination. The entire volume (21–39 ml), was fractionated and subjected to analysis providing 2 to 6 different enrichments per EpCAM-depleted sample (detailed information see S2 Table) depending on the volume. In total, 96 fractions (65x Dynabeads, 31x Adembeads) were investigated and captured cells were mounted onto glass slides. Total numbers of potential CTCs and double positive cells (Fig 5; Table 1) reflect the sum of all immunomagnetic enrichments for each blood/patient sample. Taken together, 95 potential EpCAM^neg^ CTCs (CK^pos^/CD45^neg^; mean/median: 3/2; range 1–24 CTCs) (Figs 5 and 6A), and 1069 dual-positive cells (CK^pos^/CD45^pos^; mean/median; 38/9; range 1–480) (Figs 5B and 6B) were identified (Table 2).

**Table 1:** Patient characteristics for CTCs/primary tumors and number of CK^pos^/CD45^neg^ and CK^pos^/CD45^pos^ events within the EpCAM-depleted fractions.

			CTC		primary tumor				EpCAM-depleted	
#	Pat DIII_DIV	age	CS	HER2 status	ER	PR	HER2 neu	subtype	CK^pos^/ CD45^neg^	CK^pos^/ CD45^pos^
1	DIII-1	45	1	neg	pos	pos	neg	lum A	1	15
2	DIII-2	54	0		^b^	^b^	^b^	^b^	24	18
3	DIII-3	71	147	pos	pos	pos	neg	lum A	2	11
4	DIII-4	74	0		pos	pos	neg	lum A	1	2
5	DIII-5	69	13	neg	pos	neg	neg	lum A	2	5
6	DIII-6	76	6	neg	pos	pos	neg	lum A	2	18
7	DIII-7	48	3	neg	pos	pos	neg	lum A	0	100
8	DIII-8	63	206	pos	pos	pos	neg	lum A	17	54
9	DIII-9	70	^a^/^a^		^b^	^b^	^b^	^b^	0/0	2/1
10	DIII-10	45	1	neg	pos	pos	neg	lum A	2	6
11	DIII-11	72	6	pos	pos	neg	neg	lum A	0	1
12	DIII-12	45	1/0		neg	neg	neg	basal	0/0	0/192
13	DIII-13	62	0		pos	pos	neg	lum A	3	480
14	DIII-14	58	12	pos	pos	pos	neg	lum A	0	48
15	DIII-15	38	0		pos	pos	neg	lum A	0	14
16	DIII-16	63	0		pos	pos	neg	lum A	2	13
17	DIII-17	49	^a^/^a^		neg	neg	neg	basal	3/6	2/16
18	DIII-18	75	50	neg	pos	neg	neg	lum A	8	9
19	DIII-19	54	0		pos	pos	neg	lum A	3	3
20	DIV-1	55	10	neg	pos	pos	neg	lum A	7	16
21	DIV-2	70	37	neg	pos	pos	neg	lum A	2	8
22	DIV-3	50	16	neg	pos	pos	neg	lum A	3	9
23	DIV-4	56	3	neg	pos	pos	neg	lum A	0	34
24	DIV-5	66	3	neg	pos	pos	neg	lum A	3	9
25	DIV-6	48	5/3	pos	pos	pos	neg	lum A	3/1	9/4

^a^ CTC count could not be determined

^b^ unknown

**Table 2:** Number of processed fractions and EpCAM^neg^ CK^pos^/CD45^neg^ and CK^pos^/CD45^pos^ events for each marker.

	Processed fractions	CK^pos^/ CD45^neg^	CK^pos^/ CD45^pos^
Trop2	23	27	210
CD49f	20	22	210
CK8	14	13	97
cMet	27	27	452
ADAM8	11	1	38
CD146	3	3	67
CD44	4	3	8
Hyaluronic acid	9	1	59
total	96	95	1069

Listed are the numbers of processed fractions (in total 96) for each marker as well as EpCAM^neg^ CK^pos^/CD45^neg^ and CK^pos^/CD45^pos^ events. The total number of processed samples does not include marker combinations.

Potential EpCAM^neg^ CTCs could be identified in 20 out of 29 EpCAM-depleted fractions (69%) from 25 metastatic breast cancer patients. Moreover, 64% (7 out of 11) of the samples with negative or undetermined EpCAM^pos^ CTC-status revealed at least one EpCAM^neg^ CK^pos^/CD45^neg^ event. In 5 fractions with respective EpCAM^pos^ CTCs [1 to 12 CTCs], no further potential EpCAM^neg^ CTCs could be detected. Regarding EpCAM^neg^ CK^pos^/CD45^pos^ events, only one sample (#12) did not exhibit any double positive cells, all other samples harbored at least 1 event. A high proportion of EpCAM^neg^ CK^pos^/CD45^pos^ cells was found in supernatants with corresponding low EpCAM^pos^ CTC numbers in CS (except for patient #8).

Most of the samples were enriched with antibodies against Trop2, CD49f and CK8 (63%) yielding 80% of all identified potential CTCs. All gathered CTC numbers (EpCAM^pos/neg^) as well as double positive cells can be found summarized and linked to patient characteristics in Table 1. Patients eligible for DIII/IV studies suffered from HER2-negative metastatic breast cancer, whereas for two patients (#2, #9) primary tumor characteristics could not be determined. Two patients (#12, #17) harbored triple-negative tumors (ER^-^PR^-^HER2^-^); the remaining cohort evinced primary tumors of luminal A subtype (18/21: ER^+^PR^+^HER2^-^, 3/21: ER^+^PR^-^HER2^-^).

### Molecular characterization of isolated single cells

**Fig 7:**
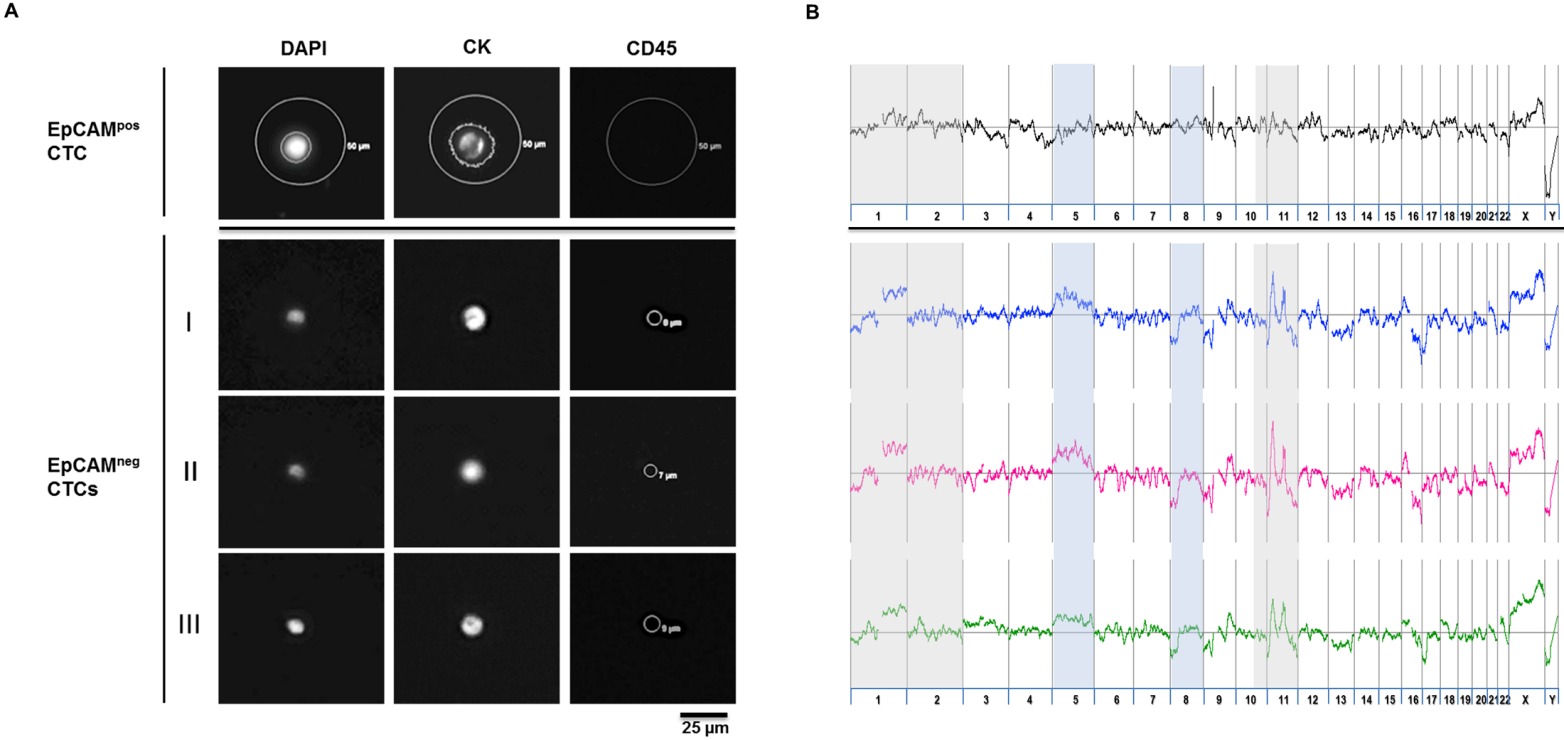
Genomic profiling of CellCelector identified and isolated whole genome amplified EpCAM^neg^ single cells confirms their malignant nature. (A) One EpCAM^pos^ CTC was selected and identified via CellSearch and re-identified with the CellCelector for a positive DAPI and CK-PE (displayed in the TRITC channel), and a negative CD45 (Cy5 channel) stain. Three EpCAM^neg^ CTCs (I, II, III) from the CellSearch EpCAM-depleted fraction of the same patient were enriched with CD44-Adembeads and stained for DAPI/pan-CK-FITC/CD45-AF647. Single cells were isolated via the CellCelector, the whole genomic material was amplified and (B) genome wide aCGH profiles were obtained confirming that EpCAM-independent enrichment captures malignant cells. Chromosomal regions highlighted in grey show common somatic copy number alterations, light blue areas represent different chromosomal aberrations between both CTC populations.

In order to validate that the isolated marker-positive cells (Fig 7A) were indeed cancer cells, we analyzed three CD44-captured EpCAM^neg^ cells in comparison to one EpCAM^pos^ CTC from the same patient for their somatic copy number profiles (Fig 7B). All four cells displayed aberrant genomes as expected for tumor cells. We found similar moving averages on chromosome 1, 2 and 11 for both EpCAM^neg^ cells and the EpCAM^pos^ CTC (Fig 7B, highlighted in grey). Besides that, EpCAM^neg^ cells showed a gain of chromosome 5 as well as a deletion at chromosome 8p (Fig 7B, highlighted in blue).

## Discussion

Until now, treatment decisions in breast cancer have been based on the expression profiles of the primary tumors. However, CTCs have been shown to display different molecular features regarding hormone receptor/EGFR and HER2 expression and thereby may elude primary treatment [37–42]. Consequently, optimized prognostic and predictive tests are required to focus on the analysis of disseminated/circulating tumor cells, which represent the actual targets of adjuvant therapies. Several methodologies for CTC enrichment have been developed that rely on EpCAM expression on CTCs. It is known that EpCAM is frequently expressed throughout different tumor entities [43, 44]. However, the loss of the EpCAM antigen has been intensively delineated [45–49]: while its up-/down-regulation has been linked to poor overall survival [50–53], its down-regulation has been described as a part of the EMT process accompanied by an enhanced migratory and metastatic potential [49, 54–56]. Thus, in order to warrant a comprehensive and systematic detection of all epithelial- and mesenchymal-like CTC subpopulations including intermediate states, the EpCAM-based enrichment and isolation techniques (e.g. by CellSearch), as the current gold standard, ought to be improved.

Here we established an integrated workflow, which captures EpCAM-depleted and hence previously unrecognized CTC subsets by using antibodies specific for cell surface proteins (Trop2, CD49f, c-Met, CK8, ADAM8, TEM8, CD44, CD47, CD146) as well as ECM components (laminin, collagen, HA).

As expected, only marginal to absent binding of MDA-MB-231 cells to EpCAM-antibodies on planar surfaces as well as coupled to beads could be observed. In contrast, efficient adhesion/capturing of EpCAM^low/neg^ cells (cell line and/or EpCAM-depleted blood fractions) could be achieved by antibodies specific for CD49f, Trop2, c-Met and CK8. By analyzing EpCAM-depleted fractions from 25 metastatic breast cancer patients, we were able to identify 22, 27, 27 and 13 EpCAM^neg^ CTCs applying enrichment for those 4 proteins. Together with targeting ADAM8, CD146, CD44 and HA, EpCAM^neg^ CTCs could be detected in 69% of all analyzed samples. For the first time we can show that EpCAM^neg^ cells captured by a CD44 antibody are of malignant nature.

In particular, CD49f (and CD146) was selected upon publications of Mostert *et al* in 2011 and 2012 [25, 26]. Herein CD49f, also designated as integrin α-6 adhesion molecule, had been implemented for a more sensitive CTC detection after a combined anti-EpCAM/CD146-enrichment. Besides its putative function as driver of metastasis [57], CD49f has been considered as stem cell marker in breast [58–60] and other solid tumors [61, 62]. In breast cancer, CD49f seems to be enriched in basal-like subtypes [63, 64], which is in concordance with our data from MDA-MB-231 showing the highest CD49f abundance, whereas its expression in luminal and HER2 subtypes was less pronounced. Trop2, a cell surface glycoprotein was implemented since it had been shown to be overexpressed in a majority of tumors [65] and to account for proliferation and invasion of tumor cells [66, 67]. Accessorily, when we started our study, Mikolajczyk *et al* had already published the use of a Trop2 (and also c-Met) antibody with regard to tumor cell enrichment via a micro-fluidic device [24]. Trop2 gained notice because it was expressed in all breast cancer cell lines examined, in contrast to EpCAM (= Trop1) expression. CD44, c-Met and CD47 were incorporated into our setup, inter alia, due to the findings of Baccelli and co-workers, who reported that CTCs possessing metastasis-initiating properties express CD44, c-Met and CD47 [28]. In a subsequent study they further showed that CD47 is a strong prognostic marker for luminal-type breast cancer patients, especially in co-expression with c-Met [68]. Our choice of CK8 was based on publications describing its cell surface expression in breast cancer cells, where it has been proposed to function as an important plasminogen binding-protein leading to increased cancer invasion [69–72]. Liu *et al* further reported that membranous CK8 might be involved in cellular protection against chemotherapeutic treatment in multi-drug resistant MCF7 cells [73]. Within our study, we could also confirm CK8 surface expression via staining of unpermeabilized MCF7 cells and subsequent flow cytometry analysis (data not shown). While cell capturing on planar surfaces using a CK8 antibody failed, CTC enrichment with anti-CK8 beads was successful. Referring to ECM components, embedded into our enrichment approach, HA emerged as the most promising candidate, at least in terms of capturing MDA-MB-231 cells. It is widely accepted that cell-matrix-interactions are pivotal for intra- and extravasation of cells and thereof for promoting metastasis [74]. HA is one of the major components of the ECM and serves as a receptor for CD44 and RHAMM (receptor for HA-mediated motility) affecting diverse cellular processes (adhesion, migration, invasion) [75–77]. The assumption that mesenchymal EpCAM^low/neg^ MDA-MB-231 cells express more CD44 and RHAMM compared to MCF7 (EpCAM^pos^, epithelial) [78] underlines our observation that invasive MDA-MB-231 cells can be captured via HA.

Apart from identifying CTCs using our defined marker setup, supernumerary EpCAM^neg^ CK^pos^/CD45^pos^ events could be detected in 28 out of 29 samples. The striking phenomenon of dual-positive (EpCAM/CK/EGFR and CD45 positive) cell detection has been observed by several groups before, while mostly being overlooked [79–82]. Consequently, the origin and occurrence of these cells, apparently combining epithelial and hematopoietic cell characteristics, still remains unclear. It has been debated that they result from fusions arising from interactions between tumor-infiltrating hematopoietic cells with epithelial cancer cells (`hemato-epithelial cells´) [83]. Other explanations might include false-positive CK^pos^ staining of leukocytes [84] or nonspecific antibody uptake owing to impaired membrane integrity. However, the fact that the number of dual-positive events is also remarkably high within the blood of healthy donors [84], and oddly, that a high proportion of those events could be assigned to patient samples with corresponding low EpCAM^pos^ CTC numbers, underscores the demand for further investigations with respect to their clinical significance.

Furthermore, the appearance of small cell clusters (2–3 cells) in EpCAM-depleted and bead-enriched fractions could be observed. This is in accordance with recent publications reporting about the detection of CTC clusters (2–50 cells), -mainly deriving from mesenchymal-like CTCs- in advanced cancers using different isolation platforms [21, 82]. Cho *et al* previously showed the presence of significant numbers of CTC aggregates in multiple epithelial cancers such as breast, prostate, lung, and pancreas [85]. Complementarily, Aceto *et al* state that these cell clusters might deduce from oligoclonal clumps of primary tumors representing a CTC subset with substantially increased metastatic capacities compared to their single counterparts in breast cancer. RNA sequencing further revealed that aggregate formation depends on the expression of plakoglobin, a member of the catenin protein family that contributes to desmosomes and adherens junctions [86, 87].

Being conscious of facing drawbacks in the light of unveiling new markers showing high specificity detection quality and quantity remain to be further studied, since those parameters, except for CK and EpCAM, are not well investigated yet. Further limitations include the unpredictability of blood sample compositions and epitope/antigen expression levels due to a high inter-patient variability. Using other markers for EpCAM-depleted patient sample analysis might have attained completely different results. This also accounts for our approach to identify EpCAM^low/neg^ cells by CK positivity: we might have missed cells which have a fully developed mesenchymal phenotype and we are aware that EpCAM^neg^/CK^neg^/CD45^neg^ cells have to be investigated further for the expression of mesenchymal markers. Additionally, in order to elucidate distinct cell surface proteins that are associated with certain tumor characteristics, the patient cohort has to be further expanded regarding sample number and tumor subtypes.

Taken together, by targeting various cell surface proteins and ECM components, we were able to optimize the enrichment of heterogeneous EpCAM^neg^ expressing cancer cells as first proof of principle, whereas the highly flexible adaptability of our approach speaks in favor for the herein presented CTC enrichment strategy. Even though coverage of all CTC subpopulations will not be utterly achieved, further characterization of these identified EpCAM^pos/neg^ and also dual-positive (CK^pos^/CD45^pos^) subsets on genomic/protein level is indispensable to determine an antibody cocktail which is evidently capturing cells of tumor origin. In the current status we cannot show clinical significance of our findings, but we were able to demonstrate the malignant nature of at least one EpCAM^neg^ subpopulation which harbors similar as well as different chromosomal aberrations in comparison to their EpCAM^pos^ counterpart. To prove clinical relevance of our approach a study identifying EpCAM^low/neg^ CTC populations with verified aberrant genomic profiles should be designed.

## Supporting Information

S1 Fig Chip/array layout, slide chemistries and binding molecules of initial cell adhesion tests. Different NEXTERION slide chemistries (AL, A+, A*star*, E, NC-C, H) were spotted with 16 arrays consisting of spots with anti-EpCAM, anti-CK8, anti-Trop2, mouse (ms) isotype (blue, #1–4, triplicates) antibodies and fibronectin (FN), poly-L-lysin, vitronectin (VN), laminin (Lam), collagen I (Col), BSA (grey, #5–10; duplicates/triplicate for FN) (0.2 mg/ml each) and tested for binding of EpCAM^pos^ cells. Adhesion was visualized by Coomassie; 2x magnification.
(TIF)

S2 Fig Total cell fluorescence of cell adhesion on multi-marker arrays. Total cell fluorescence (integral capture spot signals, in arbitrary units) of bound EpCAM^pos^ (MCF7, SKBR3, HCC1500, ZR-75-1, TMX2-28; left) and EpCAM^low/neg^ (MDA-MB-231; right) cells (**see** Fig 3) was quantified by ImageJ/Fiji 1.46. Depicted are mean values of spot signals from three separate cell adhesion experiments.
(TIF)

S1 File Recovery of SKBR3 cells by anti-EpCAM Adembeads. (DOC)

S1 Table The list of capture molecules spotted onto multi-marker arrays. (XLS)

S2 Table Overview of EpCAM-depleted samples, processed subfractions and used beads/markers per patient sample. (XLS)
