# Correction: EpCAM-Independent Enrichment of Circulating Tumor Cells in Metastatic Breast Cancer

**DOI:** 10.1371/journal.pone.0149315

**Published:** 2016-02-09

**Authors:** Helen Schneck, Berthold Gierke, Frauke Uppenkamp, Bianca Behrens, Dieter Niederacher, Nikolas H. Stoecklein, Markus F. Templin, Michael Pawlak, Tanja Fehm, Hans Neubauer, Disseminated Cancer Cell Network (DCC Net) Duesseldorf

The following information is missing from the Funding section: Funding was also received from the Anton Betz Foundation of the Rheinische Post, Duesseldorf, Germany.
