# Supplementary figures and images for: EpCAM-Independent Enrichment of Circulating Tumor Cells in Metastatic Breast Cancer
Source: PLoS One. 2015 Dec 22;10(12):e0144535. doi: 10.1371/journal.pone.0144535 (PMC4687932; doi:10.1371/journal.pone.0144535)

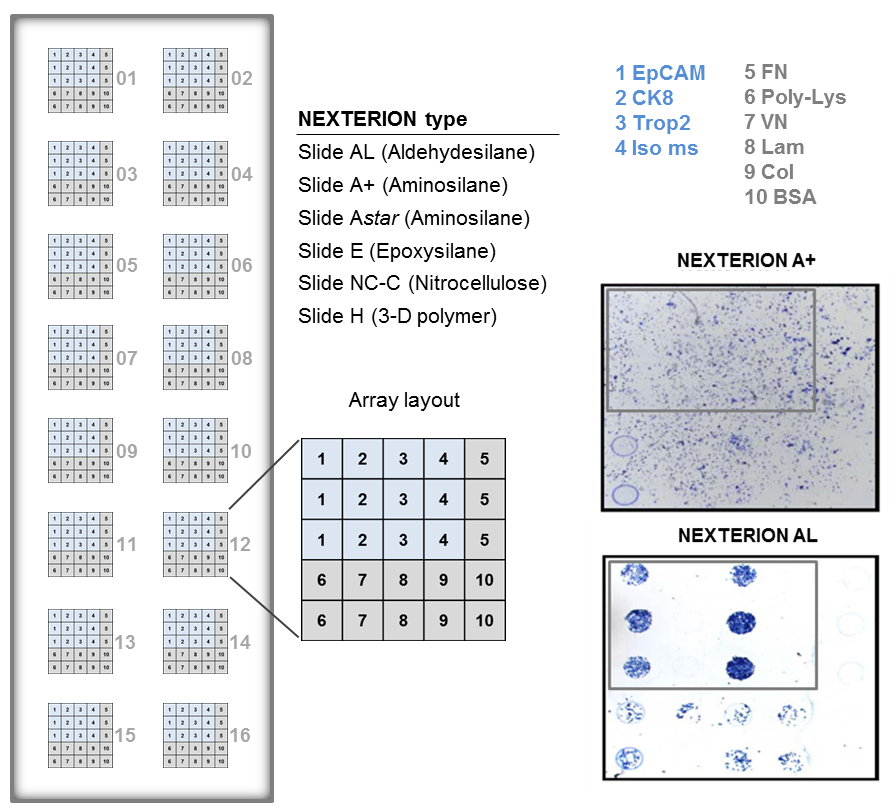

Supplement: S1 Fig — Different NEXTERION slide chemistries (AL, A+, Astar, E, NC-C, H) were spotted with 16 arrays consisting of spots with anti-EpCAM, anti-CK8, anti-Trop2, mouse (ms) isotype (blue, #1–4, triplicates) antibodies and fibronectin (FN), poly-L-lysin, vitronectin (VN), laminin (Lam), collagen I (Col), BSA (grey, #5–10; duplicates/triplicate for FN) (0.2 mg/ml each) and tested for binding of EpCAMpos cells. Adhesion was visualized by Coomassie; 2x magnification. (TIF) [file pone.0144535.s001.tif]

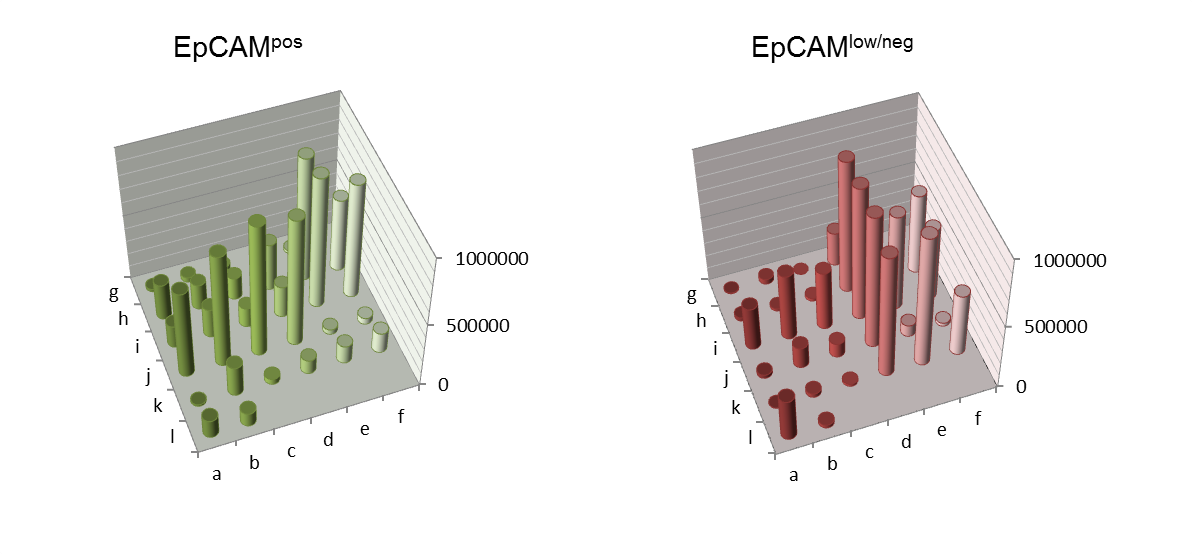

Supplement: S2 Fig — Total cell fluorescence (integral capture spot signals, in arbitrary units) of bound EpCAMpos (MCF7, SKBR3, HCC1500, ZR-75-1, TMX2-28; left) and EpCAMlow/neg (MDA-MB-231; right) cells (see Fig 3) was quantified by ImageJ/Fiji 1.46. Depicted are mean values of spot signals from three separate cell adhesion experiments. (TIF) [file pone.0144535.s002.tif]
