# Supplementary material for: EpCAM-Independent Enrichment of Circulating Tumor Cells in Metastatic Breast Cancer
Source: PLoS One. 2015 Dec 22;10(12):e0144535. doi: 10.1371/journal.pone.0144535 (PMC4687932; doi:10.1371/journal.pone.0144535)
Supplement: S1 File — (DOC) [file pone.0144535.s003.doc]

**S1 File. Recovery of SKBR3 cells by anti-EpCAM Adembeads.**

To verify successful cell isolation via Adembeads, 1x10^4^ SKBR3 cells were spiked into 2.8 % CellSave reagent/PBS and were fixed for at least 3 hours. 10, 50 and 100 cells were then incubated with anti-EpCAM Adembeads and mounted/imaged as described in the Material/Methods section. The table below shows recovery rates of 8 experiments for 10 (69%), 50 (66.8%) and 100 (69%) spiked SKBR3 cells.

| spiked SKBR3 | recovered SKBR3 | recovery |
| --- | --- | --- |
| 100 | 83 | 69.3±12 (69.3%) |
|  | 76 |  |
|  | 56 |  |
|  | 72 |  |
|  | 49 |  |
|  | 69 |  |
|  | 62 |  |
|  | 87 |  |
| 50 | 42 | 33.4±9 (66.8%) |
|  | 47 |  |
|  | 43 |  |
|  | 22 |  |
|  | 25 |  |
|  | 28 |  |
|  | 31 |  |
|  | 29 |  |
| 10 | 6 | 6.9±1 (69%) |
|  | 7 |  |
|  | 4 |  |
|  | n.d. |  |
|  | 8 |  |
|  | 7 |  |
|  | 7 |  |
|  | 9 |  |
| n.d.= cell count could not be determined | | |
